# Metabolic Score for Insulin Resistance (METS-IR) and Circulating Cytokines in Older Persons: The Role of Gender and Body Mass Index

**DOI:** 10.3390/nu14153228

**Published:** 2022-08-07

**Authors:** Virginia Boccardi, Francesca Mancinetti, Marta Baroni, Roberta Cecchetti, Patrizia Bastiani, Carmelinda Ruggiero, Patrizia Mecocci

**Affiliations:** 1 Institute of Gerontology and Geriatrics, Department of Medicine and Surgery, University of Perugia, Santa Maria della Misericordia Hospital, 06132 Perugia, Italy; 2 Division of Clinical Geriatrics, NVS Department, Karolinska Institutet Stockholm, 171 77 Stockholm, Sweden

**Keywords:** aging, inflammation, insulin, metabolism, obesity, gender

## Abstract

Background: Inflammation, along with aging processes, contributes to the development of insulin resistance (IR), but the roles of different inflammatory and other cytokines in this process remain unclear. Thus, we aimed to analyze the association between several plasma cytokines with IR as evaluated by the metabolic score for insulin resistance, METS-IR. Methods: We measured the plasma concentrations of thirty cytokines from a cohort of older persons and analyzed their role as independent factors for IR. We used regression analyses adjusted for known IR-associated factors (including age, gender, cholesterol levels, and BMI) to find the determinants of IR. Results: The study evaluated 132 subjects, mostly women (82F/50M), slightly overweight, and with a mean age of 78.5 ± 6.5 years. In the overall population, IL-15 significantly and negatively correlates with METS-IR (r = −0.183, *p* = 0.036). A regression model showed that the association between IL-15 and METS-IR was significantly modulated by gender and BMI (R^2^: 0.831). Only in women, EGF, Eotaxin and MCP-1 significantly correlated with METS-IR even after controlling by age (EGF, r = 0.250 *p* = 0.025; Eotaxin, r = 0.276 *p* = 0.13; MCP-1, r = 0.237, *p* = 0.033). Furthermore, regression models showed that these molecules were associated with METS-IR and were strongly mediated by BMI. Conclusions: Our results indicate the association between cytokines and IR has to be interpreted in a gender-specific manner. In women, EGF, Eotaxin, and MCP-1 circulating levels are associated with METS-IR being BMI a significant mediator. Understanding the role of gender in the relationship between cytokines and IR will help to define individualized preventive and treatment interventions to reduce the risk of age-related metabolic disorders.

## 1. Introduction

Aging is characterized by a deterioration in the maintenance of homeostatic processes over time, leading to functional decline and increased risk for disease and death. The aging process is characterized metabolically by changes in body composition, physiological decreases in sex steroids, and insulin resistance (IR) [1]. A state of IR is commonly observed in older adults, and a tiny connection links aging to IR: a low-grade chronic inflammation [2]. Such a state, defined as “inflamm-aging”, is characterized by the abnormal production of cytokines and the activation of pro-thrombotic and pro-inflammatory signaling pathways associated with an increased risk of diseases and death [2]. 

Molecular events underlying IR have been extensively evaluated, but the mechanism of aging-associated IR remains poorly understood. Pre-clinical studies have shown that aging per se promotes IR and inflammation in mice [3,4]. Accordingly, circulating levels of inflammatory cytokines are elevated in older persons [5,6]. Many pro-inflammatory cytokines can also indirectly induce IR by causing the induction of inflammatory genes, which then alter glucose uptake and insulin sensitivity [7]. 

Obesity, whether measured by BMI or body fat percentage, is a significant risk factor for IR and metabolic diseases [8]. Cytokines released by the adipose tissue, such as interleukin-6 (IL-6) and tumor necrosis factor-α (TNF-α), appear to directly link adiposity with insulin resistance. In humans, IR has been correlated with circulating levels of TNF-α, IL-1, IL-6, and chemokine (C-C motif) ligand (CCL) 2 in several studies [9]. Moreover, plasma levels of these cytokines are correlated with BMI and adiposity [10,11,12]. Other studies have also shown age- and gender-specific differences in IR, with increased levels in women over fifty [13].

Recently, the metabolic score for insulin resistance (METS-IR), with a higher concordance with the hyperinsulinemic-euglycemic clamp, has been developed [14]. Moreover, METS-IR is highly correlated with body fat content and can be used to quantify the degree of metabolic abnormalities in subjects while evaluating IR [14]. However, the relation between METS-IR and circulating inflammatory molecules has not yet been explored in detail, and no evidence is available in older persons. Thus, we aimed to investigate the associations between inflammatory molecules—using high-throughput techniques—and insulin resistance (by METS-IR) in older persons, also examining the effect of gender and BMI. 

## 2. Materials and Methods

### 2.1. Subjects and Study Design

The study was conducted on old age subjects referred to the Outpatient Clinic of the Department of Gerontology and Geriatrics at the University Hospital of Perugia for a general clinical evaluation. All individuals with biological and clinical evidence of inflammatory or infectious diseases, malignancies, immunologic or hematologic disorders, diabetes, or under treatment with anti-inflammatory drugs were excluded. Only subjects aged over 65 years, who were able to give written informed consent, were evaluated. Among 386 consecutively referred to our clinic, we selected 132 subjects following inclusion and exclusion criteria. All participants recruited provided informed consent, and the study adhered to the Declaration of Helsinki and was approved by the Regional Ethical Committee.

### 2.2. Clinical and Biochemical Variable Assessment

Anthropometric determinations (weight, height, and BMI) were measured using standard techniques. BMI was calculated as weight in kilograms divided by the square of height expressed in meters (kg/m^2^). Blood samples were collected in the morning after fasting overnight. Blood glucose, cholesterol, and triglycerides were analyzed using enzymatic methods, whereas high-density lipoprotein (HDL)-cholesterol was measured after isolation of low-density lipoprotein (LDL) and very-low-density lipoprotein (VLDL; Boehringer Mannheim GmbH, Germany) and LDL cholesterol was calculated using Friedewald’s method. METS-IR was determined according to the method proposed by Bello-Chavolla and colleagues [14] using the following formula: METS-IR = (ln [(2 × FPG) + TG] × BMI)/(ln[HDLc]).

### 2.3. Multiplex Assay

Fasting blood samples were collected in EDTA tubes from a peripheral vein in the morning and kept immediately on ice. Plasma was separated via centrifugation (4000 rpm for 15 min at 4 °C), aliquoted, and stored at −80 °C until analyzed. A multiplex biometric ELISA-based immunoassay was used according to the manufacturer’s instructions (MILLIPLEX MAP Human Cytokine/Chemokine Magnetic Bead Panel—Immunology Multiplex Assay, Millipore, Burlington, MA, USA). The following molecules were measured: EGF, Eotaxin, G-CSF, GM-CSF, IFN-α2, IFNγ, IL-10, IL12p40, IL12p40, IL-13, IL-15, IL-17, IL-1Ra, IL1α, IL1β, IL2, IL3, IL4, IL5, IL6, IL7, IL8, IP-10, MCP-1, MIP-1α, MIP-1β, TNFα, TNFβ, VEGF, RANTES. Measurements were performed in duplicate. The analyte concentration was calculated using a standard curve, with software provided by the manufacturer (Bio-Plex Manager Software, version 5, Bio-Rad Laboratories, Hercules, CA, USA).

### 2.4. Statistical Analyses

The observed data were normally distributed (Shapiro–Wilk W-Test) and are presented as means  ±  Standard Deviation (SD). Unpaired t-test or Pearson’s Chi-squared (χ2) test were used, as appropriate, to assess differences between groups. Simple and partial Pearson’s correlation analyses were also performed, as indicated. The independent effect of cytokines on METS-IR (dependent variable) was tested by multiple linear regression analyses controlled by potential confounding factors. The minimal sample size was estimated according to a global effect size of 25% with type I error of 0.05 and a power of 90%, resulting in 120 subjects (GPower 3.1.7). All *p* values are 2-tailed, and the level of significance was set at *p*  ≤  0.05. Statistical analyses were performed using the SPSS 20 software package (SPSS, Inc., Chicago, IL, USA).

## 3. Results

### 3.1. Sample Characteristics

The study evaluated 132 subjects, mostly women (82F/50M), slightly overweight, and with a mean age of 78.5 ± 6.5 years. Clinical and demographic characteristics of the studied population and stratified by gender are displayed in Table 1. Women had significantly higher total cholesterol, HDL, and LDL cholesterol and a lower weight than men. No difference was found in METS-IR or other characteristics between groups. METS-IR in the overall population significantly correlated with BMI (r = 0.901, *p* < 0.0001) even after controlling for gender (r = 0.907, *p* < 0.0001). No correlation was found between METS-IR and age (r = −0.130, *p* = 0.138). 

### 3.2. Plasma Cytokines and METS-IR in the Overall Population

Several cytokines including EGF (r= 0.199, *p* = 0.022), Eotaxin (r= 0.232, *p* = 0.007), IL12 p40 (r = −0.205, *p* = 0.018), IL-13 (r = −0.209, *p* = 0.016), IL-15 (r = −0.221, *p* = 0.011), IL-17 (r = −0.241, *p* = 0.005), IL-1 β (r = −0.179, *p* = 0.041), IL-4 (r = −0.174, *p* = 0.046), IL-5 (r = −0.214, *p* = 0.014), MCP-1 (r = 0.239, *p* = 0.006), MIP-1α (r = −0.216, *p* = 0.013), TNF-β (r = −0.223, *p* = 0.010) significantly correlate with BMI. In the overall population, only IP-10 (r= 0.245, *p* = 0.005) and TNF-α (r = 0.188, *p* = 0.031) significantly correlate with age. IL-15 significantly and negatively correlates with METS-IR (r = −0.183, *p* = 0.036), while a trend for EGF was found (r = 0.156, *p* = 0.07). A regression model showed that the association between IL-15 and METS-IR was significantly modulated by gender and BMI, as reported in Table 2. 

### 3.3. Plasma Cytokines, METS-IR, and Gender

Stratifying the population by gender, IL-3, IL-6, and IL-7 differed between groups, with women having lower IL-3 and higher IL-6 and IL-7 than men (Table 3). No other differences between groups were found. 

In women, EGF (r = 0.252, *p* = 0.022), Eotaxin (r = 0.365, *p* = 0.001), IL-12 p40 (r = −0.250, *p* = 0.023), IL-13 (r = −0.269, *p* = 0.014), IL-15 (r = −0.262, *p* = 0.018), IL-17 (r = −0.274, *p* = 0.013), IL-4 (r = −0.259, *p* = 0.039), IL-5 (r = −0.254, *p* = 0.021), MCP-1 (r = 0.319, *p* = 0.003), and TNF-β (r = −0.271, *p* = 0.014) significantly correlated with BMI. No significant correlation between cytokines and BMI was found in men (data not shown). In women, EGF, Eotaxin, and MCP-1 significantly correlated with METS-IR even after controlling by age (EGF, r = 0.250 *p* = 0.025; Eotaxin, r = 0.276 *p* = 0.13; MCP-1, r = 0.237, *p* = 0.033). No correlation was found in men. Regression models performed by generalized linear models in the female population revealed that EGF, Eotaxin, and MCP-1 were associated with METS-IR independent of multiple confounding variables (Table 4, Table 5 and Table 6). BMI strongly mediated all these associations. 

## 4. Discussion

Our study indicates that: (1) in a population of older persons, circulating levels of IL-15 are associated with METS-IR and mediated by BMI values; (2) in old age women, higher EGF, Eotaxin, and MCP-1 circulating levels are the main determinants for METS-IR, with BMI as a significant mediator. 

Solid evidence shows that inflammation, BMI, and IR are closely related [15,16,17]. Many pro-inflammatory cytokines are often elevated in overweight subjects with IR [18]. Recent studies also show that TNF-α, IL-6, and IL-1 promote IR, with TNF-α level as the main link between obesity and IR [19,20]. In this study, we did not observe a significant correlation between these cytokines and IR, while we found that IL-15 significantly and negatively correlated with METS-IR. 

In our population of older persons, a decrease of IL-15 by 1 unit predicted a 0.50 increase in the METS-IR. This finding suggests the potential novel protective role of IL-15 on insulin resistance. No other studies are available on humans. IL-15 is a pleiotropic cytokine that plays an important role in innate and adaptive immunity [21]. Interestingly, it has been shown that IL-15 also affects adipose tissue and induces weight loss in diet-induced obese mice [22]. Moreover, IL-15 treatment in obese mice significantly improves insulin sensitivity, glucose, and insulin responses to an oral glucose challenge [23]. More recent studies suggest that IL-15 is a myokine and is deeply involved in glucose and lipid metabolism via autocrine, paracrine, and endocrine activities [24]. In humans, plasma IL-15 is significantly decreased in obesity [25] and negatively associated with fat mass [26]. Accordingly, in our population, we found that the association between IL-15 and IR is dependent on BMI, which in turn explains 83.1% of such association. Thus, we can hypothesize that higher BMI is related to lower IL-15 levels (as reported in the correlation analysis), which is associated with higher IR evaluated by METS-IR. 

Considering that we found significant correlations between cytokines and BMI only in older women, we performed separate regression analyses by gender. We found that in older women, circulating levels of EGF, Eotaxin, and MCP-1 are the main determinants for IR, with BMI as a significant mediator. EGF levels were significantly associated with IR suggesting a causative role of EGF on insulin resistance. Again, such an association was dependent on BMI, which explained the 96.7% variability of METS-IR independent of other confounding factors (age, TC, and HDL-C). Epidermal growth factor (EGF) is a protein that stimulates cell growth, differentiation, and survival by binding to its receptor, EGFR [27]. Only a recent study has suggested a role for EGF in insulin resistance, showing an abnormal serum level in women with polycystic ovary syndrome [28]. Another study also indicates that elevation of EGF may play a role in the induction of obesity in mice after ovariectomy [29]. Further studies are necessary in humans to support our hypothesis. 

Eotaxin (or chemokine CC motif ligand 11) is an eosinophil-specific chemokine associated with the recruitment of eosinophils into sites of inflammation. We found that the increase of Eotaxin in older women predicts an increase in the METS-IR independent of multiple covariates, suggesting a causative role. However, such an association was again mediated by BMI, which explained the 96.7% variability of METS-IR independent of other confounding factors. Accordingly, previous findings show that Eotaxin is elevated in the serum of overweight patients compared with lean controls [30]. Interestingly, Eotaxin is overexpressed in visceral adipose tissue of obese subjects but is not associated with insulin resistance [31]. 

MCP-1 (Monocyte chemoattractant protein-1) is from a family of CC chemokines with a crucial role in inflammation, where it attracts or enhances the expression of other inflammatory factors [32]. MCP-1 has been implicated in the pathogenesis of numerous conditions, including cardiovascular diseases [32]. Interestingly we found that, in older women, MCP-1 levels are also associated with METS-IR values, suggesting a potential novel causative role of MCP-1 for insulin resistance. MCP-1, a member of the chemokine (chemotactic cytokine) family, may link obesity to insulin resistance [33]. In mice, MCP-1 induces insulin resistance via up-regulating the expression of *SREBP-*1*c*, a transcription factor that modulates the expression of genes related to lipid synthesis and glucose-6-phosphatase (involved in hepatic glucose production) [33]. In contrast, MCP-1 knockout mice and inhibition of MCP-1 activity exhibited improvements in insulin resistance [34]. Consistent with the findings in mice, humans show increased plasma levels of MCP-1 in type 2 diabetes mellitus [35], potentially explaining a role for IR. Interestingly, in a heterochronic parabiosis mice model, senescence of visceral adipose tissue in the old mice was diminished by sharing circulation with young animals, with a significant reduction of plasma MCP-1 [36]. 

The association between IL-15 and IR in the overall population, as well as EGF, Eotaxin, and MCP-1 exclusively in older women, disappeared when BMI was considered a confounding factor. So, it seems conceivable that the variation in systemic cytokines in IR might be a downstream factor to the increase in adiposity and that the inflamed fat tissue can produce the cytokines. Further studies need to clarify such a hypothesis. We found differences in the association between cytokines and IR only in women. The reason for the marked sex-dependent variations in this finding remains speculative. Gender differences have been reported in insulin resistance, body composition, as well as energy balance. Women have elevated general adiposity and subcutaneous adipose tissue that could explain such differences. The relationship between IR and inflammation has also been shown to have a stronger association in women than men [37]. Indeed, women seem to be exposed to a higher inflammatory burden in old age due to the peripheral reduction in the levels of estrogen sex hormones after menopause [38]. Thus, many aspects of inflammation and IR may be regulated differently between gender, influencing the predisposition to diabetes and associated metabolic disorders and complications. Our results further reinforce the need for sex-specific approaches to metabolic disorders. 

Our study has several strengths, including the relatively large number of subjects and the adjustment for multiple potential confounders. However, some limitations need to be reported. It has been conducted cross-sectionally, so it is not possible to investigate the exact effect of circulating cytokine levels on insulin resistance susceptibility. Further and more extensive studies are necessary to validate our data and better clarify the network in which cytokines are involved. Collectively, our data strongly implicate that all association studies between cytokines and IR have to be done and interpreted in a sex-specific manner and by adjusting for BMI or other measures of obesity. Understanding sex differences will help define individualized preventive and treatment interventions for IR during aging and related complications.

## Figures and Tables

**Table 1:** Demographic and clinical characteristics of the total population sample (*n* = 132), stratified by gender.

	Total Sample *n* = 132	Men *n* = 50	Women *n* = 82	*p*
Age, year	78.5 ± 6.5	86.12 ± 6.45	78.1 ± 6.9	0.436
Weight	67.2 ± 12.3	73.7 ± 10.5	63.3 ± 11.7	<0.0001
Height	159.1 ± 9.1	167.2 ± 6.8	154.2 ± 167.2	<0.0001
BMI	26.4 ± 4.2	26.3 ± 3.4	26.5 ± 4.6	0.746
Glucose (mg/dL)	102.1 ± 28.5	103.5 ± 26.6	101.2 ± 29.7	0.661
Cholesterol total (mg/dL)	205.0 ± 42.7	193.7 ± 47.4	212.0 ± 38.7	0.017
LDL-Cholesterol (mg/dL)	122.4 ± 36.9	112.8 ± 39.1	127.9 ± 34.8	0.037
HDL-Cholesterol (mg/dL)	57.7 ± 14.7	53.0 ± 14.3	60.5 ± 14.3	0.004
Triglicerides (mg/dL)	129.4 ± 57.3	131.4 ± 68.4	128.1 ± 49.7	0.749
CRP (mg/L)	0.47 ± 0.68	0.52 ± 0.92	0.45 ± 0.54	0.628
Clearance creatinine (BIS1)	67.5 ± 21.6	66.8 ± 21.6	67.9 ± 21.7	0.796
METS-IR	38.4 ± 8.0	39.2 ± 8.2	37.9 ± 7.8	0.345

BMI: Body mass index; CRP: *C*-reactive protein; METS-IR: metabolic score for insulin resistance.

**Table 2:** Linear regression analyses exploring the association of IL15 with METS-IR, controlling for multiple confounding factors in the studied population (*n* = 132).

	B	CI 95%	*p*
**Model 1**			
Age	−0.168	−0.375; 0.040	0.112
Gender	1.319	−1.487; 4.125	0.354
IL-15	−0.508	−0.990; 0.025	0.039
**Model 2**			
Age	0.110	0.019; 0.201	0.018
Gender	1.734	0.542; 2.926	0.005
IL-15	−0.090	−0.121; -0.301	0.400
BMI	1.755	1.611; 1.899	<0.0001

BMI: Body mass index. Gender indicated as: women = 0, men = 1. Model 1, R^2^ 0.057; Model 2, R^2^ 0.831.

**Table 3:** Plasmatic levels of cytokines in the studied population, stratified by gender.

	Men *n* = 50	Women *n* = 82	*p*
EGF	40.7 ± 54.0	48.6 ± 40.8	0.633
Eotaxin	142.8 ± 70.1	147.7 ± 93.6	0.749
G-CSF	35.1 ± 17.6	45.5 ± 45.2	0.092
GM-CSF	6.5 ± 2.6	7.8 ± 5.1	0.107
INF-A2	50.6 ± 14.1	54.7 ± 21.5	0.239
INF- gamma	6.7 ± 2.4	7.6 ± 4.1	0.160
IL-10	1.7 ± 1.8	2.5 ± 5.7	0.298
IL-12 p 40	9.0 ± 19.5	20.8 ± 42.0	0.066
IL-12 p 70	6.9 ± 3.5	7.3 ± 8.0	0.772
IL-13	14.2 ± 5.9	11.2 ± 5.5	0.770
IL-15	2.2 ± 1.26	2.6 ± 3.4	0.442
IL-17	8.3 ± 2.3	8.1 ± 3.7	0.730
IL-1 RA	22.6 ± 12.0	31.2 ± 15.2	0.734
IL-1 a	53.4 ±11.8	54.8 ± 27.4	0.750
IL-1 b	2.7 ± 0.8	3.1 ± 1.8	0.195
IL-2	0.9 ± 1.0	1.5 ± 3.1	0.190
IL-3	0.58 ± 0.11	0.53 ± 0.12	0.039
IL-4	10.6 ± 7.11	9.6 ± 10.2	0.568
IL-5	1.8 ± 7.3	2.2 ± 10.6	0.810
IL-6	1.4 ± 1.1	2.4 ± 3.1	0.024
IL-7	1.4 ± 1.9	2.5 ± 3.3	0.032
IL-8	4.7 ± 8.2	6.7 ± 10.8	0.251
IP-10	722.8 ± 425.5	821.2 ± 454.1	0.221
MCP-1	391.0 ± 154.4	435.3 ± 271.8	0.295
MIP-1 a	3.6 ± 6.8	5.2 ± 9.2	0.289
MIP-1 b	22.6 ± 17.2	24.8 ± 23.2	0.559
TNF-a	18.0 ± 10.3	21.1 ± 16.1	0.237
TNF-b	11.5 ± 5.2	15.9 ± 7.0	0.705
VEGF	155.0 ± 54.3	197.5 ± 30.8	0.337
RANTES	37008 ± 38721	38278 ± 33646	0.843

Cytokines are expressed in pg/mL.

**Table 4:** Linear regression analyses exploring the association of EGF (Epidermal Growth Factor) with METS-IR, controlling for multiple confounding factors in women (*n* = 82).

	B	CI 95%	*p*
**Model 1**			
Age	−0.192	−0.415; 0.030	0.089
TC	0.012	−0.030; 0.054	0.561
HDL-C	−0.235	−0.345; −0.126	<0.0001
EGF	0.016	0.001; 0.030	0.037
**Model 2**			
Age	0.091	0.042; 0.140	<0.0001
TC	0.000	−0.009; 0.009	0.956
HDL-C	−0.165	−0.189; −0.142	<0.0001
EGF	−0.001	−0.004; 0.002	0.571
BMI	1.532	1.457; 1.607	<0.0001

TC: total cholesterol; HDL-C: HDL cholesterol; BMI: body mass index. Model 1, R^2^ 0.257; Model 2 R^2^ 0.967.

**Table 5:** Linear regression analyses exploring the association of Eotaxin with METS-IR, controlling for multiple confounding factors in women (*n* = 82).

	B	CI 95%	*p*
**Model 1**			
Age	−0.184	−0.396; 0.027	0.087
TC	0.018	−0.022; 0.058	0.374
HDL-C	−0.273	−0.378; −0.168	<0.0001
Eotaxin	0.029	0.130; 0.450	0.001
**Model 2**			
Age	0.092	0.040; 0.141	<0.0001
TC	0.000	−0.009; 0.009	0.956
HDL-C	−0.163	−0.187; −0.139	<0.0001
Eotaxin	−0.001	−0.005; −0.003	0.522
BMI	1.537	1.458; 1.616	<0.0001

TC: total Cholesterol; HDL-C: HDL Cholesterol; BMI: body mass index. Model 1, R^2^ 0.328; Model 2, R^2^ 0.967.

**Table 6:** Linear regression analyses exploring the association of MCP-1 (Monocyte Chemoattractant Protein-1) with METS-IR, controlling for multiple confounding factors in women (*n* = 82).

	B	CI 95%	*p*
**Model 1**			
Age	−0.164	−0.384; 0.055	0.139
TC	0.011	−0.029; 0.052	0.578
HDL-C	−0.256	−0.364; −0.149	<0.0001
MCP-1	0.008	0.002; 0.111	0.007
**Model 2**			
Age	0.090	0.042; 0.139	<0.0001
TC	0.000	−0.008; 0.009	0.940
HDL-C	−0.164	−0.188; −0.141	<0.0001
MCP-1	0.000	−0.002; 0.001	0.522
BMI	1.535	1.458; 1.611	<0.0001

TC: total cholesterol; HDL-C: HDL cholesterol; BMI: body mass index. Model 1, R^2^ 0.285; Model 2 R^2^ 0.967.

## Data Availability

The data presented in this study are available on request from the corresponding author. The data are not publicly available due to privacy.

## References

[1] Barzilai N., Huffman D.M., Muzumdar R.H., Bartke A. (2012). The critical role of metabolic pathways in aging. Diabetes.

[2] Sanada F., Taniyama Y., Muratsu J., Otsu R., Shimizu H., Rakugi H., Morishita R. (2018). Source of Chronic Inflammation in Aging. Front. Cardiovasc. Med..

[3] He W., Yuan T., Choezom D., Hunkler H., Annamalai K., Lupse B., Maedler K. (2018). Ageing potentiates diet-induced glucose intolerance, β-cell failure and tissue inflammation through TLR4. Sci. Rep..

[4] Moreno-Fernandez M.E., Sharma V., Stankiewicz T.E., Oates J.R., Doll J.R., Damen M.S.M.A., Almanan M.A.T.A., Chougnet C.A., Hildeman D.A., Divanovic S. (2021). Aging mitigates the severity of obesity-associated metabolic sequelae in a gender independent manner. Nutr. Diabetes.

[5] Brüünsgaard H., Pedersen B.K. (2003). Age-related inflammatory cytokines and disease. Immunol. Allergy Clin. North Am..

[6] Bruunsgaard H., Pedersen M., Pedersen B.K. (2001). Aging and pro-inflammatory cytokines. Curr. Opin. Hematol..

[7] Shoelson S.E., Lee J., Goldfine A.B. (2006). Inflammation and insulin resistance. J. Clin. Investig..

[8] Martinez K.E., Tucker L.A., Bailey B.W., LeCheminant J.D. (2017). Expanded normal weight obesity and insulin resistance in US adults of the national health and nutrition examination survey. J. Diabetes Res..

[9] Makki K., Froguel P., Wolowczuk I. (2013). Adipose Tissue in Obesity-Related Inflammation and Insulin Resistance: Cells, Cytokines, and Chemokines. ISRN Inflamm..

[10] Tataranni P.A., Ortega E. (2005). A burning question: Does an adipokine-induced activation of the immune system mediate the effect of overnutrition on type 2 diabetes?. Diabetes.

[11] Das A., Mukhopadhyay S. (2012). The Evil Axis of Obesity, Inflammation and Type-2 Diabetes. Endocr. Metab. Immune Disord. Drug Targets.

[12] Dandona P., Aljada A., Bandyopadhyay A. (2004). Inflammation: The link between insulin resistance, obesity and diabetes. Trends Immunol..

[13] Gayoso-Diz P., Otero-Gonzalez A., Rodriguez-Alvarez M.X., Gude F., Cadarso-Suarez C., García F., De Francisco A. (2011). Insulin resistance index (HOMA-IR) levels in a general adult population: Curves percentile by gender and age. The EPIRCE study. Diabetes Res. Clin. Pract..

[14] Bello-Chavolla O.Y., Almeda-Valdes P., Gomez-Velasco D., Viveros-Ruiz T., Cruz-Bautista I., Romo-Romo A., Sánchez-Lázaro D., Meza-Oviedo D., Vargas-Vázquez A., Campos O.A. (2018). METS-IR, a novel score to evaluate insulin sensitivity, is predictive of visceral adiposity and incident type 2 diabetes. Eur. J. Endocrinol..

[15] Chait A., den Hartigh L.J. (2020). Adipose Tissue Distribution, Inflammation and Its Metabolic Consequences, Including Diabetes and Cardiovascular Disease. Front. Cardiovasc. Med..

[16] Artemniak-Wojtowicz D., Pyrżak B., Kucharska A.M. (2020). Obesity and chronic inflammation crosslinking. Cent. Eur. J. Immunol..

[17] Ellulu M.S., Patimah I., Khaza’ai H., Rahmat A., Abed Y. (2017). Obesity & inflammation: The linking mechanism & the complications. Arch. Med. Sci..

[18] Schmidt F.M., Weschenfelder J., Sander C., Minkwitz J., Thormann J., Chittka T., Mergl R., Kirkby K.C., Faßhauer M., Stumvoll M. (2015). Inflammatory cytokines in general and central obesity and modulating effects of physical Activity. PLoS ONE.

[19] Rehman K., Akash M.S.H. (2016). Mechanisms of inflammatory responses and development of insulin resistance: How are they interlinked?. J. Biomed. Sci..

[20] Al-Mansoori L., Al-Jaber H., Prince M.S., Elrayess M.A. (2022). Role of Inflammatory Cytokines, Growth Factors and Adipokines in Adipogenesis and Insulin Resistance. Inflammation.

[21] Lodolce J.P., Burkett P.R., Koka R.M., Boone D.L., Ma A. (2002). Regulation of lymphoid homeostasis by interleukin-15. Cytokine Growth Factor Rev..

[22] Shi J., Fan J., Su Q., Yang Z. (2019). Cytokines and Abnormal Glucose and Lipid Metabolism. Front. Endocrinol..

[23] Barra N.G., Chew M.V., Holloway A.C., Ashkar A.A. (2012). Interleukin-15 treatment improves glucose homeostasis and insulin sensitivity in obese mice. Diabetes Obes. Metab..

[24] Pedersen B.K., Febbraio M.A. (2012). Muscles, exercise and obesity: Skeletal muscle as a secretory organ. Nat. Rev. Endocrinol..

[25] Barra N.G., Reid S., MacKenzie R., Werstuck G., Trigatti B.L., Richards C., Holloway A.C., Ashkar A.A. (2010). Interleukin-15 contributes to the regulation of murine adipose tissue and human adipocytes. Obesity.

[26] Nielsen A.R., Hojman P., Erikstrup C., Fischer C.P., Plomgaard P., Mounier R., Mortensen O.H., Broholm C., Taudorf S., Krogh-Madsen R. (2008). Association between interleukin-15 and obesity: Interleukin-15 as a potential regulator of fat mass. J. Clin. Endocrinol. Metab..

[27] Herbst R.S. (2004). Review of epidermal growth factor receptor biology. Int. J. Radiat. Oncol. Biol. Phys..

[28] Gao J., Song Y., Wang D., Zhang D., Huang X. (2021). Serum Levels of PDGF, EGF, and sFlt-1 in Patients with Polycystic Ovary Syndrome and Their Predictive Effects on Pregnancy Outcomes. Evidence-Based Complement. Altern. Med..

[29] Kurachi H., Adachi H., Ohtsuka S., Morishige K.I., Amemiya K., Keno Y., Shimomura I., Tokunaga K., Miyake A., Matsuzawa Y. (1993). Involvement of epidermal growth factor in inducing obesity in ovariectomized mice. Am. J. Physiol. Endocrinol. Metab..

[30] Herder C., Haastert B., Müller-Scholze S., Koenig W., Thorand B., Holle R., Wichmann H.E., Scherbaum W.A., Martin S., Kolb H. (2005). Association of systemic chemokine concentrations with impaired glucose tolerance and type 2 diabetes: Results from the cooperative health research in the region of augsburg survey S4 (KORA S4). Diabetes.

[31] Vasudevan A.R., Wu H., Xydakis A.M., Jones P.H., Smith E.O.B., Sweeney J.F., Corry D.B., Ballantyne C.M. (2006). Eotaxin and obesity. J. Clin. Endocrinol. Metab..

[32] Singh S., Anshita D., Ravichandiran V. (2021). MCP-1: Function, regulation, and involvement in disease. Int. Immunopharmacol..

[33] Kanda H., Tateya S., Tamori Y., Kotani K., Hiasa K.I., Kitazawa R., Kitazawa S., Miyachi H., Maeda S., Egashira K. (2006). MCP-1 contributes to macrophage infiltration into adipose tissue, insulin resistance, and hepatic steatosis in obesity. J. Clin. Investig..

[34] Nio Y., Yamauchi T., Iwabu M., Okada-Iwabu M., Funata M., Yamaguchi M., Ueki K., Kadowaki T. (2012). Monocyte chemoattractant protein-1 (MCP-1) deficiency enhances alternatively activated M2 macrophages and ameliorates insulin resistance and fatty liver in lipoatrophic diabetic A-ZIP transgenic mice. Diabetologia.

[35] Ahmed S.F., Shabayek M.I., Abdel Ghany M.E., El-Hefnawy M.H., El-Mesallamy H.O. (2018). Role of CTRP3, CTRP9 and MCP-1 for the evaluation of T2DM associated coronary artery disease in Egyptian postmenopausal females. PLoS ONE.

[36] Ghosh A.K., O’Brien M., Mau T., Qi N., Yung R. (2019). Adipose Tissue Senescence and Inflammation in Aging is Reversed by the Young Milieu. J. Gerontol. Ser. A Biol. Sci. Med. Sci..

[37] Cartier A., Côté M., Lemieux I., Pérusse L., Tremblay A., Bouchard C., Després G.P. (2009). Sex differences in inflammatory markers: what is the contribution of visceral adiposity?. Am. J. Clin. Nutr..

[38] Duarte A.C., Hrynchak M.V., Gonçalves I., Quintela T., Santos C.R.A. (2016). Sex Hormone Decline and Amyloid β Synthesis, Transport and Clearance in the Brain. J. Neuroendocrinol..

